# Tactics employed by healthcare providers in Denmark to determine the vaccination needs of asylum-seeking children: a qualitative study

**DOI:** 10.1186/s12913-018-3661-1

**Published:** 2018-11-14

**Authors:** Cathrine S. Nakken, Marie Norredam, Morten Skovdal

**Affiliations:** 1 Danish Research Center for Migration, Ethnicity and Health, Section for Health Services, Research, Department of Public Health, University of Copenhagen, Øster Farimagsgade 5, 1353 København K, Denmark; 2 Section of Immigrant Medicine, Department of Infectious Diseases, Copenhagen University Hospital, Hvidovre Hospital, Hvidovre, Denmark

**Keywords:** Refugee children, Asylum-seeking children, Immunization, Vaccination, Healthcare workers, Tactics, Denmark

## Abstract

**Background:**

Many asylum-seekers to Denmark come from war-torn countries where conflict and insufficient health care infrastructures disrupt vaccine programmes and result in very few children and their families presenting documentation of vaccinations on their arrival in asylum-centers. There is a need to explore how healthcare providers, in the absence of vaccine documentation, determine the vaccination needs of newly arrived refugee children.

**Methods:**

To explore the tactics employed by healthcare professionals who screen and vaccinate asylum-seeking children in Denmark, we conducted semi-structured interviews between December 2015 and January 2016 with six healthcare professionals, including three doctors and three public health nurses. The interviews were digitally recorded, transcribed and subjected to a thematic network analysis.

**Results:**

The analysis revealed that healthcare providers adopt a number of tactics to ascertain children’s immunization needs. They ask into the children’s vaccination history through the use of qualified interpreters; consult WHO lists of immunization programmes worldwide; draw on tacit knowledge about country vaccination programmes; consider the background of parents; err on the side of caution and revaccinate.

**Conclusions:**

This is one of the first studies to demonstrate the tactics employed by healthcare providers to ascertain the immunization needs of asylum-seeking children in a western receiving country. The findings suggest a need for clear guidance at a national level on how to determine the vaccination needs of asylum-seeking children, and an international effort to secure reliable immunization documentation for migrant populations, for example through virtual immunization records.

## Background

European countries have in recent years experienced a large influx of asylum-seekers as a result of conflicts and civil unrest in parts of North Africa, the Middle East and Central Asia. According to the European Commission, over 1.3 million asylum applicants were registered in Europe in 2015. An estimated 30% of all asylum-seekers are children, of whom nearly a quarter are unaccompanied [1]. In Denmark alone, a little more than 21,000 refugees applied for asylum in 2015, an increase of 44% compared to the previous year [2]. Although the number of asylum-seekers has plummeted in recent years, the sudden influx of migrants and refugees in the run up to 2015 offers a unique opportunity to critically examine the preparedness of European health services in responding to the health needs of asylum-seekers. One health response pertains to vaccinations. The World Health Organization (WHO) [3] states that asylum-seekers and migrants should be vaccinated as soon as possible according to the vaccination program of the country in which they intend to settle in. Vaccination initiatives targeting child asylum-seekers are important because young children are at particular risk of not having completed their vaccination course because of war and conflict-related interruptions to the vaccination programmes in their home countries as well as lack of access to immunization programs while on the run/migrating [4].

All asylum seekers to Denmark are upon their arrival accommodated in a reception asylum center north of Copenhagen, and later referred to other asylum centers. It is the reception asylum center, which is run by Red Cross Denmark that is primarily responsible for the vaccination of asylum-seeking children. Within 10 days of arrival, a voluntary health assessment is offered at the health clinic of the reception asylum center. Children (< 18 years of age) are assessed by a nurse and a doctor separately. In accordance with current guidelines of the National Board of Health [5], asylum-seekers should be vaccinated according to the national routine immunization schedule. However, asylum-seeking families only very rarely carry documentation of their immunization history [6, 7], and rarely recall what vaccines their children have received in the past [8]. Against this background, we explore how healthcare providers determine the vaccination needs of asylum-seeking children.

To do this we frame the actions of healthcare providers as ‘tactics’, taking inspiration from de Certeau’s [9] notions of ‘strategies and tactics’. Strategies refer to the direction offered by people or organizations in positions of power, such as the State. This may include funding cuts, policy changes, national and international guidelines, or the absence thereof. Tactics on the other hand may refer to the everyday acts and occurrences that healthcare providers adapt in order to exhume a bit of control, or make do, in a setting deemed constraining. By drawing on de Certeau’s notion of ‘tactics’, we can disentangle the bundle of ‘doings’ that healthcare providers – in the context of broader political, cultural and economic forces – adapt in their everyday routines in order to cooperate, resist or deal with factors constraining their ability to determine the vaccination needs of asylum-seeking children.

## Methods

To understand how healthcare professionals ascertain the immunization needs of asylum-seeking children we employed an exploratory qualitative interview study design. We opted for an interview study design because it allowed us to flexibly and exploratory examine their experiences, perspectives and accounts of how they determined the vaccination needs of asylum-seeking children. The study was registered with the Danish Data Protection agency and followed their ethical guidelines. Informed consent was obtained from all participants, who in turn were assured confidentiality.

### Study setting and participants

At the start of 2017, there were 45 asylum centers in Denmark run by the Red Cross or different municipalities. These include a reception center, two departure centers, eight children centers, and one ‘special care’ center [10]. In 2015, we invited three public health nurses and three medical doctors (age 45–65) working at two Red Cross asylum centers, including the reception center, to participate in the study. The participants worked at centers in the Zealand area of Denmark and were identified via a gatekeeper who was an employee stakeholder. All those invited to participate in the study agreed to do so. As per our inclusion criteria, the healthcare providers, as part of their day-to-day routine of offering health services to asylum seekers, screened and vaccinated asylum-seeking children arriving in Denmark. Apart from one participant who had only recently been employed (but who had broad experience within the same field in the public sector), all had extensive prior experience of working for the Red Cross. None of the participants were previously known to the researcher. We made a conscious decision of only recruiting six healthcare workers, as only a very small number of healthcare providers routinely screen and vaccinate asylum-seeking children in Denmark. If we recruited many more, it would be difficult to ensure anonymity. Our six participants effectively make up a significant and representative proportion of healthcare workers routinely vaccinating asylum-seeking children.

### Data collection and analysis

The interviews took place during the months of December 2015 and January 2016 and were conducted face-to-face at the two asylum centers. The interviews were structured by a topic guide collaboratively developed by the authors. We devised questions by drawing on thematic categories presented by Mulrow et al. [11] and Ozbolt and Faan [12] who highlight materials (e.g., policies and guidelines), competences (e.g., understanding of patient’s situation) and meanings (e.g., ethics) as core elements entering into clinical decisions. We ended up with 14 questions, covering topics such as information availability, cultural and language barriers to communication, resource availability, the use of decision making tools, time availability and age-based access concerns’.

The interviews lasted an average of 45 min and were conducted by CN (a native Norwegian speaker) in a mix of Norwegian-Danish. Due to the similarities between Norwegian and Danish, this mixing of the two languages did not appear to impact the flow of the interviews.

CN subsequently translated and transcribed the interviews into English and used the ‘table method’ for coding text segments within the transcripts [13]. This involved, firstly, transferring text segments into word document tables, and secondly, printing, cutting out and placing the codes, which make up basic themes, on an actual table for an exploration of networked themes [13, 14]. This involved CN and MS working together to cluster the 12 emerging basic themes into six more interpretive organizing themes. This process of cross case analysis, enabled us to move beyond description of individuals’ accounts and their individualized personal experiences, and instead map out and condense some of the more prevalent experiences and perceptions as reported by the informants. As not all the basic and organizing themes are of relevance to this article, and because the strategies devised by our participants to determine the vaccination needs of asylum-seeking children emerged as a significant finding, we proceeded with a secondary and more interpretive interrogation of our emerging themes, aided by our theoretical framework. This process led to a new thematic network, with nine of our basic themes contributing to five new global themes (which make up the structure of our results) and one global theme (see Table 1).

**Table 1:** Thematic network: from codes to global theme

Codes and basic themes	Organising themes	Global theme
• Communication obstacles • Feelings and experiences around the use interpreters	Tactic 1: Asking into their vaccination history through the use of qualified interpreters	Tactics employed by healthcare providers to determine the vaccination needs of asylum-seeking children
• Use of information ‘tools’	Tactic 2: Consulting WHO lists of immunization programmes worldwide	
• Lack of vaccination documentation • Limited time available for screening and vaccinations	Tactic 3: Drawing on tacit knowledge about country vaccination programmes	
• Cultural differences and barriers • Information given from parents	Tactic 4: Considering the background of parents	
• Feelings around current procedures • Doctor’s individual decision	Tactic 5: Err on the side of caution and revaccinate	

## Results

Asylum-seeking children presenting to Red Cross Asylum centres are subject to routine statutory health screening. This involves, first, an appointment with a public health nurse, who talks to the child and accompanying adults about their medical history. The nurse documents everything. After seeing the public health nurse, the children will be referred to a doctor for further examinations and vaccinations as necessary. The doctors have the final say in determining whether a child needs vaccinations. At one center, it was only the doctors who vaccinated, while at another center, the public health nurses also administered vaccinations. Both the public health nurses and the doctors expressed difficulty in ascertaining the vaccination needs of asylum-seeking children, stating that almost no one came with documentation of their vaccinations on arrival, relying on their memory and self-reported accounts of vaccinations. Elsewhere we have reported that nearly a third of asylum-seeking children and adolescents in Denmark were in need of further vaccinations [15]. We now present findings on some of the tactics these healthcare professionals adopted – in the absence of documentation – to decide what children to vaccinate, and allude to some of the challenges of these tactics.

### Tactic 1: asking into their vaccination history through the use of qualified interpreters

They all stated that the use of interpreters played an important role in enabling the communication between themselves, the children and their families. All agreed that it was important to have access to an interpreter who could be physically present and who was knowledgeable about the medical field. One doctor said it was expected that the interpreters would know medical expressions and vaccine names, and not ask the families if their children had received the ‘blue looking vaccination’ against the disease that ‘gives red dots’ and so on.

“Well, it is expected that the interpreters know the terms and can interpret sufficiently. And it shouldn’t be at the level where they ask ‘have you gotten the red vaccine?’ or ‘the blue medicine’ or... that’s not good enough... they are supposed to know the terms and expressions. And I presume that the parents get the information on what it is we are doing, like ‘now he is vaccinated against...’ and such, right? I presume that.” -Doctor, Red Cross.

Some of the public health nurses stated that they preferred interpreters to be physically present, but that they are now expected to cut costs and use phone interpreters. This implied different challenges, for example, if the families came late and the interpreter was scheduled for other calls, there would not be time to assess their vaccination needs. There could also be a lot of noise when families with many children were in the office, which made it difficult to hear the interpreter speaking on the phone. These challenges were often compounded, if the families spoke local dialects that the interpreters were not familiar with. A doctor also mentioned that it was also quite challenging to translate the vaccination documentation over the phone, since he, or the family, could not read and pronounce the words correctly.

Nonetheless, all of the healthcare professionals mentioned that most of the interpreters they had encountered did a professional and important job as their spokespersons at the asylum centers. A public health nurse mentioned that they occasionally encountered interpreters of sub-standard; however, she now had the experience to be able to deal with such challenges.

“The interpreters are very professional. I think the ones we work with are good ones. Of course, we have had interpreters who were not good, but then we do something about it. So I feel it’s working out, and I also begin to understand a bit of what they say, because after a while of listening to a language one learns to hear if they say something that sounds familiar or something that shouldn’t be. So, I believe they are professional interpreters and they function as my spokespersons*.”* –Public health nurse, Red Cross.

### Tactic 2: consulting WHO lists of immunization programmes worldwide

The healthcare professionals all had access to the World Health Organization’s list of immunization programmes worldwide on their computers, and could easily check each country’s immunization program, thereby determining what the child should have received according to his/her age, and what vaccinations the child would be lacking and would therefore need to receive in Denmark.

“We use it a lot! I have saved it in my favorites. I use it all the time. It is really useful, what would I do without it?” –Public health nurse, Red Cross.

One doctor said that he also used information from outbreak reports to learn more about specific vaccination needs. For example, if there had been an outbreak of polio in Syria, he expressed that he would be more observant and vaccinate the children arriving in Denmark against polio.

“If there is a five year old child coming from Syria, then I can go into the Syrian vaccination program that I will find on the WHO list, and then I can assess what the child is supposed to have had and match it with the Danish vaccination program. It makes it much easier from this point on.”–Doctor, Red Cross.

### Tactic 3: drawing on tacit knowledge about country vaccination programmes

Two of the other doctors and public health nurses said that their assessment would depend on where the children came from and their family situation. One doctor added that children from some countries were almost never vaccinated, while others were well vaccinated.

“It also depends on where they come from. Syrian children are normally very well vaccinated for example. And then it also depends a bit on how they act here with me. Like, how long have they been travelling, and why have they chosen to flee and seek asylum in Denmark. -We cannot do more than ask them!” -Doctor, Red Cross.

### Tactic 4: considering the background of parents

One public health nurse said that she had the impression that educational status and vaccination needs were linked, assuming that if the caretakers were well educated, the chances that the child would be vaccinated before coming to Denmark and not require further vaccinations were higher. Another nurse expressed the opinion that it also depended on geographical variations within the countries and hierarchical position. For example, if the family lived in a rural area and had little access to health services, or if they were considered entitled or not to receive healthcare in their country, that might affect whether or not they had received vaccinations prior to leaving their country of origin.

“I think it relates to how well educated the people from the area they come from are. I think it is linked, the education status of their population and their vaccination status. I think it’s related.” –Public health nurse, Red Cross.

### Tactic 5: err on the side of caution and revaccinate

If the children were unaccompanied and the vaccination needs difficult to determine, the doctors stated that they would almost always start to revaccinate, to ensure that the child would be ‘covered’. One doctor said that in his experience, doctors handled these situations differently. Some preferred to start vaccinating from the beginning again, while others preferred to give a booster vaccine, depending on the child’s age. Another doctor also expressed that the decisions regarding what to do in these cases were based on each doctors’ individual assessment and experience. According to one doctor’s statement, all unaccompanied children who were coming were at least 15 years old, and would therefore know a little bit about their own vaccination situation, which made it easier.

“(…) But the unaccompanied that come, they are 15 years old minimum. I haven’t met someone who is younger than that. So they normally know something about it, and if they don’t then they receive a booster.”-Doctor, Red Cross.

Another doctor stated that he thought the unaccompanied minors were the most difficult to assess, as they almost never remembered their vaccination status, regardless of age, and even if they did remember being vaccinated, they did not know which ones.

“I would say that the unaccompanied minors can be difficult. Also because I’m a bit careful starting up from the beginning with them, I know that they probably have had some boosters in other countries. Now, it’s not particularly dangerous to give them one more, but one can easily over-give and give them lots of vaccines that they might have had already. So I think they are difficult, because they can almost never remember if they have received anything at all.” –Doctor, Red Cross.

In the absence of asylum-seekers bringing with them some sort of vaccination documentation, the healthcare providers generally agreed that these tactics worked reasonably well, but also felt that they could be more disciplined and better at investigating the children’s immunization status. One doctor stipulated that current tactics probably resulted in many children getting ‘over-vaccinated’.

“Well, I’m not in doubt that one could be better disciplined and investigate more thoroughly. But now it is like this, and so they just get boosters, maybe even if my gut feeling tells me that they are actually finished with their vaccinations. Personally, I think that might be a kind of ‘overkill’ in a way. But I have the impression that it works quite well, and that there aren’t many who ‘slip through’ and are not vaccinated at the asylum centers.” –Doctor, Red Cross.

## Discussion

We set out to explore the tactics employed by healthcare providers in Denmark to ascertain the vaccination needs of asylum-seeking children. The first tactic pertained to obtaining documentation of previous vaccinations. However, more often than not, this was not available, instigating a whole range of tactics, including asking into their vaccination history through the use of qualified interpreters; consulting WHO lists of immunization programmes worldwide; drawing on tacit knowledge about country vaccination programmes; considering the background of parents; err on the side of caution and revaccinate. These practices demonstrate the efforts made by healthcare providers to try and do what they see as right within the limited time and resources they have at their disposal: 1) develop a full picture of children’s vaccination history; and 2) revaccinate in situations of uncertainty.

The lack of immunization documentation has been shown to be an important influence on the immunization assessments of healthcare professionals’ in other contexts [8, 16–18]. Paxton et al.’s [8] study found that parent-reported vaccination status did not predict serological immunity, indicating that the legitimacy of the information provided is questionable. Similarly, another study found that in depending on statements given by families, healthcare professionals did not feel completely certain of the legitimacy of the information given which made the assessments of vaccination status of refugee children more complex [19]. Additionally, some have argued that knowledge held by healthcare providers regarding a child’s immunization status did not predict with certainty the vaccination status of the child, and advocate for the need of formal documentation as the secure way of determining a child’s immunization status [19]. In this study we found that in cases of uncertainty regarding the information provided, administering boosters was considered the preferred, low-risk solution of healthcare providers. However, others propose serology testing in cases of doubt as the favored solution [19]. Although the findings in this study point to booster vaccines as the preferred option among healthcare staff, there are numerous studies that have found a correlation between the overly frequent administration of certain vaccines and increased risks of negative reactions [18, 20], which brings this practice as the most desirable option into question. Others have discussed the cost and practicality of serology testing where findings suggest that the cost-effectiveness and practice of serology testing is debatable [18]. Poethko-Müller et al. [21] found that healthcare staff may overestimate vaccination coverage in cases where formal documentation is not available. An interesting addition to this debate is Chai et al.’s [16] study, which found that although there was a risk of overestimating vaccination needs because of lost documentation among healthcare professionals, the percentage of missing vaccinations among refugee children was similar to that of children who remained in Ethiopia -the country of origin of the majority of the population investigated in their study. Their discovery indicates that pre-migration factors in the children’s country of origin are superior in determining immunization outcomes than post-migration factors, as exemplified by the tendency of healthcare professionals in the receiving country to overestimate vaccination coverage in the event of a lack of formal documentation.

Our findings are constrained by some methodological limitations, which deserve mentioning. First, the study relies on self-reported data and it is difficult for us to ascertain what happens in clinical practice. Future research in this area of study may consider adopting an ethnographic approach Second, and relatedly, the participants were all recruited through a gatekeeper and represent an international relief organization whose reputation they may feel obliged to protect. As such, our study was susceptible to social desirability bias, such as participants representing themselves as following official Red Cross policies. CN only noted a few instances where participants appeared cautious about sharing their perspectives. Third, our study was cross-sectional and only provides a brief snapshot (during the peak of the migration flow to Denmark) into healthcare providers’ strategies for determining the vaccination needs of asylum-seeking children at a particular moment in time. Longitudinal research, unpacking the dynamic and changing nature of their tactics would be useful. Fourth, the generalizability of our findings is limited and may not apply to other settings, as the structure and delivery of health services for asylum-seeking children in our setting may well vary from others. This study only explored the perspectives of healthcare providers. Future research could usefully broaden its scope and include the perspectives and experiences of refugee families.

## Conclusions

This is one of the first studies to demonstrate the tactics employed by healthcare providers to ascertain the immunization needs of asylum-seeking children in a western receiving country. The study uncovers several areas in need of attention. First, health care providers appear to need more information and clear guidance to help them determine the vaccination status of asylum-seeking children and to reach a more homogenous and systematic assessment amongst providers. The Danish Red Cross has some internal guidelines but no guidelines exist from the National Board of health on this issue. This is further complicated by the fact that asylum seekers may be promptly resettled in one out of the 98 municipalities in Denmark, or in asylum centers run by local municipalities. Studies show that municipalities have very diverse and often inadequate services regarding the health reception of refugee families [22]. Second, our study expands on the importance of healthcare professionals having accurate information and immunization documentation to determine vaccination status and needs. The contradicting arguments presented in this article indicate that the information available for clinical decision-making is not optimal, and highlights the need for joint and international efforts to secure reliable immunization documentation for migrant populations. Improved recordkeeping would contribute to a decrease in the incidence of over**-**vaccination without adversely affecting immunity. WHO encourage governments to provide caregivers with documentation of the vaccines given to avoid unnecessary vaccination. Even if this practice was widespread, the circumstances forcing people to flee their home countries and seek asylum in another country, makes reliance on a paper-based immunization documentation system untenable. In light of our increasingly globalized world and digital age, perhaps we should begin to consider virtual immunization records?

## Abbreviations

WHO: World Health Organization
